# Client perception of service quality at the outpatient clinics of a General hospital in Lagos, Nigeria

**DOI:** 10.11604/pamj.2015.22.68.6228

**Published:** 2015-09-28

**Authors:** Babatunde Enitan Ogunnowo, Tolulope Florence Olufunlayo, Salami Suberu Sule

**Affiliations:** 1 Department of Community Health and Primary Care, College of Medicine of the University of Lagos, Nigeria; 2 National Postgraduate Medical College of Nigeria, Nigeria

**Keywords:** Client perception, service quality, outpatient clinics

## Abstract

**Introduction:**

Service quality assessments have assumed increasing importance in the last two decades. They are useful in identifying gaps in services been provided with the ultimate aim of guaranteeing quality assurance. The objective of this study was to assess the client perception of service quality at the outpatient clinics of Randle General hospital, Lagos.

**Methods:**

A descriptive cross sectional study was conducted from March to May 2013. A multistage sampling technique was used to select respondents and data was collected with the aid of modified SERVQUAL questionnaires. The data was analysed with aid of EPI-INFO 2002 and statistical significance was set at a P value 0.05 for statistical significance.

**Results:**

Total of 400 respondents were interviewed. The mean age was 40 years with a standard deviation of 15.2 yrs. The highest mean score of 4.35 out of a possible maximum of 5 was recorded in assurance domain while the lowest mean score of 4.00 was recorded in the responsiveness domain. The overall mean score of all the domains was 4.20 with standard deviation of 0.51. Overall majority (80.8%) of respondents rated the overall service quality as good/ very good. After linear regression, the assurance domain was the most important predictor of the overall perceived service quality (p< 0.001).

**Conclusion:**

The overall perceived service quality was good. The major deficiencies were in the responsiveness domain and especially the waiting time. The hospital management should implement measures to improve the responsiveness of services by ensuring prompt delivery of services.

## Introduction

Service delivery refers to the systematic arrangement of activities in service giving institutions with the aim of fulfilling the needs and expectations of service users and other stake- holders with optimum use of resources [1]. Quality in healthcare service delivery refers to services that meet set standards, implying excellence and satisfy the needs of both consumers and healthcare practitioners in a way that adds significant meaning to both parties [2]. In the last three decades, assessment of quality of care has assumed increasing importance [3, 4]. Quality of care is important as it influences the utilization of the services [5, 6], compliance with treatment [7, 8] and ultimately health outcome [9, 10]. Quality of care can be accessed from the perspective of the clients, service providers and managers of institution [11]. Assessment of client perception of quality of care and satisfaction with services has assumed a more prominent role in the last two decades especially with the advent of consumer movement organizations in developed countries [12]. It provides a feedback about services rendered highlighting areas of strengths as well as deficiencies that need to be improved upon. Service quality is a measure of the degree of discrepancy between consumer's perception and expectations. Consumer dissatisfaction occurs when the expectations are greater than the actual performance of service delivered by the organization. In contrast, clients have high degree of satisfaction when the perception of service is clearly in excess of expectations [13]. Surveys from various parts of the world have shown varying degrees of client overall satisfaction with health services in developed and developing countries. The Rate of satisfaction from the surveys ranges from 22% to 95% [14, 15]. Many of these surveys have identified various problems with quality of service in hospitals such as long waiting times, overcrowding at clinics, poor attitude of staff and lack of drugs, among others [16, 17]. In Nigeria, the health care system is organised at three level; primary, secondary and tertiary. The primary health care services are provided by local government in primary health centres, secondary care services by state governments in general hospitals while the federal government provides tertiary care in teaching hospitals [18]. Surveys in Lagos have shown that just like in many parts of the world 60%-70% of population utilise private health facilities for their health needs [19]. One of the reasons for the lower level of utilisation of public health facilities compared to private ones is the poor perception of the quality of services in government hospitals [20]. Periodic assessment of client perception of service quality is crucial to identify area of critical need for improvement as well as to provide a baseline to assess the effect of interventions to improve quality of care in the spirit of quality assurance [21]. Hospitals exist to provide service to clients and as such providing high quality of care should be of topmost priority as clients are major stakeholders in health care delivery. The Randle general hospital Surulere is a secondary level facility in Lagos. It provides outpatient and inpatient services to residents of Lagos. It has a mission to provide prompt and affordable health care services to all patients in a clean and healthy environment. There is a need to determine how well or otherwise, the hospital has been able to achieve its mission statement. The outpatient clinic is the gateway to almost all of hospital service and globally 80% of clients in hospital are attended to at the outpatient department [22]. It therefore implies that the findings from surveys on quality of care at outpatient clinics will be a reflection of the quality of care in the entire hospital. Indeed better outpatient services are the keys to health gains for the poor [23]. Few studies have been carried out to assess the client perception of service quality at the outpatient clinics of the hospital. Findings of this study will provide baseline data on the current situation regarding quality of care as perceived by clients. It will also identify areas of strengths and deficiencies in services provided as perceived by the clients. The information obtained from the study will be useful in designing interventions aimed at improving the overall quality of care at the outpatient clinics in the hospital. This is especially important in the view of the high premium played on continuous quality Improvement and total quality management in recent years [24]. The objective of this study was to assess the client perception of service quality at the outpatient clinics of Randle general hospital, Surulere, Lagos.

## Methods

### Study area

Lagos state is one of the 36 states in Nigeria. It is located in the South Western zone of the Nigeria. It is bounded on the North and the East by Ogun state in the West by Republic of Benin and on the south by the Atlantic ocean. It has a population of 17 million and has 20 local government areas LGA and 37 local council development area LCDA [25]. Surulere local government is one of the twenty local government areas. In Lagos State, Randle General Hospital is located at Number 66 Randle Avenue in Surulere local government area of Lagos state. Originally a health centre donated to the federal government of Nigeria by the united African company at independence in 1960, it was taken over by the Lagos state government in 1999 and was upgraded to a general hospital in 2001. It provides outpatient and inpatient services in the general outpatient clinics, medical, surgical, Paediatric accident and emergency clinics, dental, DOTS, and obstetric and gynaecological clinic and the relevant wards The maternal and child health services are provided at the Maternal and child care centre Gbaja which was moved out of the main hospital at Randle Avenue and commenced operation in 2011. The hospital has a staff strength of 405 and a bed capacity of 156. It has an X -ray department, pharmacy, laboratory and blood bank. There are 18 departments in the Hospital in 2011, there were a total of l06, 120 Outpatient visits reported in various clinics of the hospital

**Study design:** descriptive cross sectional study to assess client perception of service quality at the outpatient clinics of Randle General Hospital Lagos.

**Study population:** clients attending the outpatient clinics of the Randle general hospital Surulere.

**Sample size determination:** sample size was calculated using the formula for descriptive studies [26].

N= (Z2 PQ)/d2; where N = minimum sample size; Z= Critical value corresponding to 95% confidence level= 1.96; P= Proportion with parameter (client satisfied with service = 50% assumption); Q= 1-p; D= precision

So N= (1.96)2* 0.5* 0.5/ (0.05)^2^

N= 3.84 X 0.25/0.0025

N= 384

The minimum required sample size is 384. However a total sample size of 400 was used for the study

### Sampling methodology

A multi stage sampling method was used for the study. In stage one, five (5) outpatient clinics were selected from the various outpatient clinics. The selected clinics were the medical outpatient clinic, surgical outpatient clinic, general outpatient clinic, paediatric outpatient clinic and Obstetrics and Gynaecological clinics. In stage two, A systematic sampling method was used to select respondents in the selected clinics. The number of clients to be selected in each clinic was determined by proportionate allocation based on the statistics for the previous year. In each clinic, A sampling interval was calculated at each clinic based on the average clinic attendance.

### Data collection tool and procedure

A modified SERVQUAL questionnaire was adapted for the study. The instrument has been validated for use in the health sector [27]. The SERVQUAL framework utilises five criteria in assessing service quality specifically Tangibles, Reliability, responsiveness, assurance and empathy [28]. Tangibles- refers to the physical facilities, equipment and appearance of personnel Reliability refers to the ability to perform promised service dependably and accurately Responsiveness refers to the willingness to help consumers and provide prompt service Assurance or security is the knowledge and courtesy of employees and their ability to inspire trust and confidence. Empathy- caring, individualised attention provided to consumers. The questionnaire had four sections. Section A documented the socio demographic characteristic of respondents, Section B documented some aspects of the process such as waiting time, consultation time while section C documented the client perception of various domain of service quality based on SERVQUAL tool. The instrument was pretested at General hospital Mushin and necessary corrections were effected. The instrument was administered by two (2) research assistants as exit interviews. The assistants were experienced research assistants with a minimum of Bachelor's degree in Social sciences and they were trained over a one day period through role plays and, demonstration to ensure that high quality data is collected. Data collection took place over a period of three weeks in April 2013. Each clinic was visited twice a week so as to get an appropriate representation of patients attending the clinics. On selected days in each clinic, the first patient was selected at random while subsequent patients were selected based on the calculated sampling interval. Selected patients were interviewed at the end of clinic consultation as exit interviews by the research assistants.

### Data management

The data collected was entered and analysed with EPI-INFO 2002 version 3...5...4. Windows Results were presented in tables and figures. The mean of the various domains of service quality was calculated. Linear regression was used to determine which domain of service quality was most important contributor to the overall client perception of service quality.

### Ethical considerations

Ethical approval was obtained from the health research ethics committee of the Lagos University teaching hospital. Permission for the study was obtained from the management of the hospital. Informed consent was obtained from the client attending the clinic. The confidentiality of information collected was secured by restricting access to the data collected to investigator and research assistants. Anonymity of the clients was ensured by not including the personal details of the clients in the instrument. Client were assured that their responses will not be used against them and it will not influence the care they will receive in the facility.

## Results

A total of 400 respondents were interviewed in various clinics of Randle general hospital. Regarding the socio-demographic characteristics of respondents, slightly more than quarters (26%) of the respondents were aged 30-39 years while only 3% were below 20 years. The mean age was 40 yrs with a standard deviation of 15.2years. Majority (78.7%) of respondents were female while almost two thirds (64%) were Christians. Almost half (47.2%) of respondents had secondary education while slightly more than half (52%) of them were in occupations that can be classified as unskilled. Almost two fifth (39.5%) of respondents in the study were from those attending the general outpatient clinic and more than half (51%) had visited the clinics in the hospital at least three times. Majority (52.7%) of respondents spent more than three hours before they saw the doctor while only 1.2% spent less than 30 mins before seeing the doctor. More than one third of respondents (41%) estimated that they spent more than 15minutes with the doctor. Table 1 shows the respondents perception of the tangible domain of service quality. Majority (60.7%) of respondents agreed that the clinic they were attending was clean. Similarly majority (59.8%) of respondents agreed that the clinic had a comfortable environment. Majority (54.5%) of the respondents were uncertain about the clinic having information brochure about their activities. Almost half (47.5%) of respondents strongly agreed that privacy was observed when given care and 56.5% of respondents agreed that the staff were neat in appearance. More than one third (38.5%) of respondents strongly agreed that there were well maintained medical facilities in the clinic On a scale of 1 to 5 with strongly disagree on 1 and strongly agree on 5, the mean score for the clinic is clean statement was 4.30 ± 0.63, while that for the statement the clinic had a comfortable environment was 4.33 ± 0.60. The mean score for the statement the clinic had an information brochure was 3.50 ± 0.83 while the mean score for privacy been observed when given care was 4.27± 0.84. With regards to staff been neat in appearance, the mean score was 4.41 ± 0.52. The mean score for the statement, there are well maintained facilities in the clinic was 4.05 ± 0.85. Table 2 shows the respondents perception of reliability domain of service quality. Slightly more than one- third (38%) of respondents agreed that services were provided at appointed time. Majority (58.7%) of respondents agreed that services were carried out right the first time. Majority (61.5%) of respondents strongly agreed that the doctors were professional and competent. Similarly majority (62.8%) of respondents agreed that there was fast retrieval of documents in the clinics. Slightly less than half (46%) of respondents agreed that there was consistency of service charges in the clinics in the hospital. The mean score for the statement services were provided at appointed time was 3.90± 0.82 while for services been carried out right the first time was 4.37 ± 0.55. The mean score for the statement doctors are professional and competent was 4.54 ± 0.66. Regarding the statement there is fast retrieval of document, the mean score was 3.85 ± 0.66. The mean score for the statement there is consistency of service charges was 4.16 ± 0.96.

**Table 1:** Respondents perception of tangible domain of Service quality

Variable	Strongly disagree (N%)	Disagree (N%)	Uncertain (N%)	Agree (N%)	Strongly agree (N%)	Total (N%)	Mean score	Standard deviation
The clinic is clean	2(0.5)	9(2.3)	3(0.7)	243(60.7)	143(35.8)	400(100)	4.30	0.63
The clinic has a comfortable environment	0(0)	9(2.3)	2(0.5)	239(59.8)	150(37.4)	400(100)	4.33	0.60
The clinic has information brochure	0(0)	21(5.3)	219(54.5)	94(23.5)	67(16.7)	400(100)	3.50	0.68
Privacy is observed when given care	1(0.3)	15(3.8)	49(12.2)	145(36.2)	190(47.5)	400(100)	4.27	0.84
Staff are neat in appearance	0(0)	0(0)	6(1.5)	226(56.5)	168(42.0)	400(100)	4.41	0.52
There are well maintained medical facilities in the clinic	0(0)	3(0.7)	129(32.3)	114(28.5)	154(38.5)	400(100)	4.05	0.85

OVERALL MEAN: 4.16; OVERAL L STANDARD DEVIATION: 0.54

**Table 2:** Respondents’ perception of reliability domain of service quality

Variable	Strongly disagree (N%)	Disagree (N%)	Uncertain (N%)	Agree (N%)	Strongly agree (N%)	Total (N%)	Mean score	Standard deviation
Services are provided at appointed time	3(0.7)	3(0.7)	139(34.8)	152(38.0)	103(25.8)	400(100)	3.90	0.82
Services are carried out right the first time	1(0.3)	3(0.7)	4(1.0)	235(58.7)	157(39.3)	400(100)	4.37	0.55
Doctors are professional and competent	1(0.3)	2(0.5)	27(6.7)	124(31.0)	246(61.5)	400(100)	4.54	0.66
There is fast retrieval of document	0(0)	12(3)	86(21.5)	251(62.8)	51(12.8)	400(100)	3. 85	0.66
There is consistency of service charges	1(0.3)	21(5.3)	71(17.7)	123(30.7)	184(46.0)	400(100)	4.16	0.96

OVERALL MEAN: 4.18; OVERALL STANDARD DEVIATION: 0.57

Table 3 shows the respondents perception of responsiveness domain of service quality. Almost half (49.5%) of respondents strongly agreed that clients are given prompt service. Slightly more than half (50.7) of respondents strongly agreed that doctors are responsive to client needs. Similarly 51% of respondents agreed that nurses were responsive to client needs. Majority (58.5%) of respondents strongly agreed that the attitude of doctors instil confidence in clients while 42.7% of them agreed that attitude of nurses instil confidence in clients. Almost half of respondents (47.5%) were uncertain regarding the statement that the waiting time does not exceed one hour while 35.5% of them disagreed with the statement. The mean score for the statement clients are given prompt service was 4.39 ± 0.73 while that for the statement doctors are responsive to client needs was 4.43 ± 0.66. The mean score for the statement nurses are responsive to client needs was 4.35± 0.79. Similarly the mean score for the statement attitude of doctors instil confidence in clients was 4.54 ± 0.62 while that for the statement attitude of nurses instil confidence in clients was 4.04 ± 0.10. Regarding the statement waiting time does not exceed one hour, the mean score was 2.78 ± 0.75. Table 4 shows the assurance domain of service quality. Majority (51.2%) of the respondents strongly agreed that doctors are courteous and friendly while 44.2% of respondents strongly agreed that nurses are courteous and friendly. Only 37.2% of respondents strongly agreed that the doctors possess a wide spectrum of knowledge. Majority (56.2%) of respondents strongly agreed that clients were treated with dignity and respect. Similarly 51.7% of respondents agreed that clients get explanation thoroughly about their medical condition The mean score for the variables in assurance domain was lowest for the statement nurses are courteous and friendly at 4.16 ± 0.92 and highest for the statement clients are treated with dignity and respect at 4.49 ± 0.70 Table 5 shows the empathy domain of service quality. Only 42.2% of the respondents strongly agreed that feedback is obtained from clients. Majority (52.5%) of respondents strongly agreed that doctors have the best interest of clients at heart while 42% of them strongly agreed that nurses have the best interest of patients at heart. Majority (52.5%) of respondents agreed that doctors understand specific needs of patients. The mean score for the variables under the empathy domain ranged from 4.9 ± 0.79 for the statement nurses have the best interest of clients at heart to 4.46± 0.64 for the statement doctors understand specific needs of patients. Only about one-third (38.0%) strongly agreed that the charges in the clinic in the hospitals were affordable while majority (57.5%) agreed that the clinic is easily accessible Overall, majority (72.5%) of the respondents perceived that the overall service quality was good while 18.6% perceived that the overall service quality was average. Only (0.8%) perceived that the overall service quality was poor. Linear regression showed that assurance domain was the most important predictor of perceived overall service quality in the hospital as shown in Table 6.

**Table 3:** Respondents perception of responsiveness domain of service quality

Variable	Strongly disagree (N%)	Disagree (N%)	Uncertain (N%)	Agree (N%)	Strongly agree (N%)	Total (N%)	Mean score	Standard deviation
Patients are given prompt service	2(0.5)	8(2.0)	27(6.8)	165(41.2)	198(49.5)	400	4.39	0.73
Doctors are responsive to client needs	1(0.3)	3(0.7)	25(6.3)	168(42.0)	203(50.7)	400	4.43	0.66
Nurses are responsive to client needs	2(0.5)	9(2.3)	41(10.2)	144(36.0)	204(51.0)	400	4.35	0.79
Attitude of doctors instil confidence in clients	1(0.3)	8(2.0)	3(0.7)	154(38.5)	234(58.5)	400	4.54	0.62
Attitude of nurses instil confidence in clients	4(1.0)	24(6.0)	63(15.8)	171(42.7)	138(34.5)	400	4.04	0.10
Waiting time does not exceed one hour	6(1.5)	142(35.5)	190(47.5)	57(14.2)	5(1.3)	400	2.78	0.75

OVERALL MEAN: 4.00; OVERALL STANDARD DEVIATION: 0.60

**Table 4:** Respondents’ perception of the assurance domain of service quality

Variable	Strongly disagree (N%)	Disagree (N%)	Uncertain (N%)	Agree (N%)	Strongly agree (N%)	Total (N%)	Mean score	Standard deviation
Doctors are courteous and friendly	3(0.7)	11(2.8)	12(3.0)	169(42.3)	205(51.2)	400(100)	4.41	0.74
Nurses are courteous and friendly	1(0.3)	26(6.5)	58(14.5)	139(34.7)	176(44.0)	400	4.16	0.92
Doctors possess wide spectrum of knowledge	1(0.3)	1(0.3)	15(3.7)	234(58.5)	149(37.2)	400	4.33	0.57
Clients are treated with dignity and respect	3(0.8)	8(2.0)	4(1.0)	160(40	225(56.2)	400	4.49	0.70
Clients get explanation thoroughly about their medical condition	7(1.8)	14(3.5)	18(4.5)	154(38.5)	207(51.7)	400	4.37	0.86

OVERALL MEAN SCORE: 4.35; OVERALL STANDARD DEVIATION: 0.58

**Table 5:** Respondents’ perception of the empathy domain of service quality

Variable	Strongly disagree (N%)	Disagree (N%)	Uncertain (N%)	Agree (N%)	Strongly agree (N%)	Total (N%)	Mean score	Standard deviation
Feedback is obtained from clients	1(0.3)	4(1.0)	37(9.3)	189(47.2)	169(42.2)	400	4.31	0.70
Doctors have clients best interest at heart	1(0.3)	3(0.7)	23(5.8)	210(52.5)	163(40.7)	400	4.33	0.64
Nurses have clients best interest at heart	2(0.5)	10(2.5)	47(11.8)	172(43.0)	169(2.2)	400	4.29	0.79
Doctors understand specific needs of patients	1(0.3)	4(1.0)	15(3.7)	170(42.5)	210(52.5)	400	4.46	0.64

OVERALL MEAN SCORE: 4.33; OVERALL STANDARD DEVIATION: 0.61

**Table 6:** Multiple linear regression of overall perception of service quality in outpatient clinics of Randle General hospital based on domains of service quality

Domains of service quality	Coefficient	standard error	F-TEST	P-value
Tangibles	-0.140	0.085	2.723	0.101
Reliability	- 0.056	0.104	0.297	0.587
Responsiveness	0.139	0.091	2.343	0.126
Assurance	0.327	0.096	11.634	0.001
Empathy	0.111	0.096	1.342	0.247
Affordability and Accessibility	-0.071	0.059	1.421	0.234

## Discussion

This study utilized the modified SERVQUAL Questionnaire to assess service quality in Randle general hospital, Lagos Nigeria. This study has provided the opportunity to identify areas of strengths and weakness in quality of health care provided in the hospital. The socio-demographic findings show that majority of clients were females and had at least a secondary education. The average age of respondent was also 40 yrs. This finding is consistent with that from other studies [29, 30]. The Service quality was assessed using the five SERVQUAL dimensions of Tangibles, Reliability, responsiveness, assurance, empathy and an additional dimension of affordability and accessibility. The overall mean score of Tangibles dimensions was 4 16 out of a possible 5 with a standard deviation of 0.54. This generally suggests that the clinics had a good and conducive environment. However, the lowest score on the tangibles domain was with regards to availability of brochure about the clinic facilities. This is an area of weakness that can be improved upon by the hospital management through the production of brochure and information pamphlets detailing the activities in each specific clinic. The brochure should also include client duties and client rights, information on safety in the hospital and contact details in case of safety issues, map of the hospital and information on strategic locations. This is despite the fact that the hospital is a public one which unlike private hospitals may not place a high premium on the marketing of the available services. The Overall mean score in the reliability domain was 4.18 with a standard deviation of 0.57 implying a good performance. The lowest scores of 3. 85 was recorded on the issue of how fast documents are retrieved and whether services are provided at the appointed time. This areas need to be improved upon by the management through an assessment of the current processes in the record section of the hospital with a view to identifying ways to reduce the time spent in the retrieval of documents such as case files. The overall mean score for the responsive domain was 4. 00 with a standard deviation of 0.60 which implies an overall good performance. The lowest score of 2.78 was recorded on the issue of waiting time not exceeding 1hour. This implies a long waiting time which needs to be addressed through client flow analysis to identify the areas with the greatest delay and the required interventions to reduce it. The highest score of 4.54 was recorded on how the attitude of doctors instils confidence in the clients. This is a positive development that should be encouraged and sustained. With regards to the assurance domain, the overall mean score was 4.35 with a standard deviation of 0.58. All the items in the domain had mean score above 4.0 with implies a good performance. The findings should be sustained to ensure that clients are assured of a high level of quality healthcare delivery. The overall mean score for the empathy domain was 4.33 with a standard deviation of 0.61. This implies that clients perceived that health workers had the best interest of clients at heart and understood the specific needs of the client. The mean score for the affordability domain was 4.04 while that for the accessibility domain was 4.56 which implies that clients perceived that services were affordable and the clinics were accessible. Overall, the mean service quality across all domains was 4.20 with a standard deviation of 0. 51. Other studies have assessed service quality with various tools and methodologies which makes comparison with this study inappropriate [31, 32]. The study showed that assurance domain was the most important predictor of overall service quality. This implies that hospitals must place a high premium on this domain in order to achieve optimal service quality. This study however has its limitations. The study focused only on outpatients so the findings cannot be generalized to inpatients. Secondly, as with most quality assessment studies, courtesy bias is a possible limitation to finding.

## Conclusion

In conclusion, this study, which utilised the modified SERVQUAL tool, showed that clients perceived the service quality at the outpatients clinic of Randle General Hospital to be good. The highest score was recorded in the assurance domain while the lowest score was recorded in the responsive domain. The assurance domain was the most important factor influencing the overall perceived service quality in the outpatient clinics. The hospital management need to prioritize interventions to improve the responsiveness of the services provided in the hospital in order to further improve the service quality in the hospitals.
